# Artificial intelligence in ultrasound: divergent pathways, shared challenges

**DOI:** 10.1093/bjrai/ubag015

**Published:** 2026-08-26

**Authors:** Viksit Kumar, Michael Blaivas

**Affiliations:** AutonomUS Medical Technologies, Cambridge, MA, 02139, United States; Department of Medicine, University of South Carolina School of Medicine, Columbia, SC, 29209, United States

**Keywords:** artificial intelligence, deep learning, ultrasonography, point-of-care ultrasound, foundation models, medical education, image segmentation, computer-aided diagnosis

## Abstract

Artificial intelligence (AI) in ultrasound is often reviewed as a unified field, though in practice it spans 3 distinct deployment environments: clinical radiology (traditional imaging) ultrasound, point-of-care users, and ultrasound education. The 3 environments share the same underlying AI methods but differ in image quality, operator skill, time allotted to scan, regulatory exposure, and acceptable failure modes. In clinical radiology ultrasound, FDA-cleared decision support is routine for breast and thyroid lesion characterization, obstetric biometry, and cardiac quantification, and emerging in liver, vascular, and interventional imaging. In point-of-care ultrasound, AI enables bedside clinicians to acquire diagnostic images under real-time guidance and to document examinations that would otherwise go unrecorded. In education, AI reshapes competency assessment and scales instruction. This article offers the practicing radiologist and imaging specialist a framework for matching AI to deployment context, names the mature applications in each pathway, and addresses cross-cutting challenges (data, validation, regulation, bias, liability, reimbursement, environmental cost) that every deployment must navigate. The recurring message: treating “ultrasound AI” as a monolith is a repeated error, and honest attention to deployment context matters most.

## Introduction and purpose

Ultrasound holds a peculiar position in diagnostic imaging. Once best known for its impact in low-resource settings where few other imaging options were available, ultrasound has since become a modality of first choice for an expanding range of applications across radiology, obstetrics, cardiology, emergency medicine, anesthesiology, and critical care.^1^ Overwhelmed radiology departments struggle to keep up with an unending surge in demand for imaging services, while specialties historically unfamiliar with ultrasound have discovered its potential at the bedside and now struggle to train colleagues new to ultrasound and assure competency.^2^^,^^3^ The result is a single modality being pulled in 2 directions at once: toward increasingly pressured radiology departments, and toward a rapidly growing but internally fragmented bedside-ultrasound community. Ultrasound training, caught between these 2 worlds, forms a third.

Artificial intelligence (AI) is widely invoked as the solution to all 3 pressures, faster reads, safer bedside scans, and scalable instruction. The reality, as the sections below develop, is more nuanced. The same AI capabilities behave very differently when deployed into each of these settings, and a tool that performs well in one pathway can fail, sometimes unsafely, in another. This article is written for the radiologist and imaging specialist encountering ultrasound AI in practice, whether evaluating a commercial tool, working with a point-of-care ultrasound (POCUS) program, or weighing which published results apply to their own department. Its purpose is not to exhaustively survey the ultrasound AI literature, several high-quality reviews already exist, but to provide a practical framework that keeps deployment context at the center of every decision.^4^^,^^5^

Three recommendations anchor the article: First, recognize that “ultrasound AI” describes 3 different fields, and match the AI task to the pathway; second, read the published evidence with deployment in mind, not algorithmic novelty; third, know the regulatory, legal, and environmental setting within which any deployment will live. The sections that follow expand each in turn.

This article is a narrative review, not a systematic one. Literature was identified through searches of PubMed, Scopus, and Google Scholar through July 2026, combining ultrasound terms (ultrasound, ultrasonography, echocardiography, point-of-care ultrasound) with artificial-intelligence terms (deep learning, machine learning, foundation model, segmentation), supplemented by hand-searching of reference lists, the FDA 510(k) and De Novo databases, and regulatory guidance documents. Preference was given to prospective, multicenter, or externally validated studies and to FDA-cleared or CE-marked systems; single-center retrospective work is cited where it represents the best available evidence and is identified as such. No formal inclusion/exclusion protocol was applied, consistent with the article’s purpose of providing a deployment framework rather than an exhaustive evidence synthesis.

## How artificial intelligence works in ultrasound, and where it breaks

Ultrasound AI is better understood by task than by algorithm family. High-quality reviews describe the specific architectures in use, convolutional networks, recurrent networks, transformers, diffusion models, but those labels tell the reader comparatively little about what the processor is actually being asked to produce. It is more useful to name the task categories that ultrasound AI applications actually perform. Three broad categories cover nearly all clinical applications. **Image classification** assigns an image or cine loop to a learned category, a liver, an apical 4-chamber view, a probable malignancy.^6^  **Object detection** draws bounding boxes around structures of interest, the gallbladder, a nerve, a lymph node, and is the workhorse of real-time acquisition guidance. **Image segmentation** traces a mask around a structure and is responsible for the identity of every pixel inside it; this task underlies automated biometry, ejection-fraction estimation, and quantitative measurement generally. Concrete, named examples of each category appear throughout this review and in Table 1: EchoNet-Dynamic performs segmentation-based ejection-fraction estimation, Koios DS performs classification-based lesion risk stratification, and ScanNav Anatomy performs real-time detection of nerve-block anatomy. Capability is task- and domain-specific: a model that excels on natural images or text, including today’s general-purpose vision-language models, has no demonstrated or regulatory claim to ultrasound interpretation unless it has been trained and validated on ultrasound data for that specific task.

**Table 1:** Representative FDA-cleared and CE-marked AI-enabled ultrasound devices across 16 clinical applications.

Clinical domain	Product (manufacturer)	Regulatory clearance	Primary task/output	Representative evidence
**Breast**	Koios DS Breast (Koios Medical)	FDA 510(k) K190442 (2019), K242130 (Nov 2024); CE mark	Lesion characterization with BI-RADS alignment	Irmici 2025 *Eur J Radiol*: largest diagnostic gain for junior reader across 226 lesions
**Thyroid**	Koios DS Thyroid (Koios Medical); MEDO-Thyroid (MEDO.ai/Exo)	FDA 510(k) K242130 (2024); MEDO 510(k) K203502 (April 2021); CE mark	TI-RADS-aligned nodule risk stratification; sweep-based volume and nodule analysis	Ashton 2024 Thyroid: AUC 0.70 standalone, 0.76 combined with radiologist; 17% FNA reduction across 649 nodules
**Obstetric biometry**	GE Voluson with SonoLyst/fetalHQ; Samsung BiometryAssist; Philips SmartExam OB	FDA 510(k) as part of cart systems (eg, Voluson K231965, 2023); CE MDR	Automated fetal biometry (BPD, HC, AC, FL); ISUOG 20-view recognition	Vendor validation studies; Pokaprakarn 2022 *NEJM Evid* (gestational age from blind sweeps)
**Obstetric anomaly screening**	Sonio Detect + Sonio Suspect (Sonio SAS)	FDA 510(k) (Detect K230365, 2023; Suspect K243614, Feb 2025); CE MDR	Anatomy-survey view completeness (Detect); detection of 8 fetal anomalies (Suspect)	Sonio Suspect reader study: radiologist AUC 0.69-0.91 with AI
**Gestational age (low-resource)**	Butterfly Gestational Age Tool (Butterfly Network)	FDA 510(k) (2026, first blind-sweep AI for GA)	Gestational age from non-expert blind-sweep acquisition	Pokaprakarn 2022 *NEJM Evid* (research prototype underpinning the clinical tool)
**Cardiac: acquisition guidance**	Caption AI/Caption Guidance (Caption Health/GE HealthCare)	FDA De Novo DEN190040 (Feb 2020); CE mark	Real-time view-quality feedback; transthoracic echo for non-specialist users	Narang 2021 *JAMA Cardiol*: 98.8% LV, 92.5% RV diagnostic across 240 patients by nurses with no prior echo experience
**Cardiac: acquisition guidance (multi-system)**	UltraSight (UltraSight)	FDA 510(k) (July 2023; expanded multi-device clearance K251416, December 2025)	AI-guided echo acquisition across handheld, laptop, and cart systems	Vendor clinical studies; first cleared AI echo-guidance across all form factors
**Cardiac: full quantification**	Us2.v1/Us2.v2 (Us2.ai)	FDA 510(k) (v1 2021, v2 2024); CE mark	45 automated cardiac parameters including volumes, EF, strain, and chamber quantification	Ouyang 2020 *Nature* (EchoNet-Dynamic, field benchmark): EF MAE 4.1%, HFrEF AUC 0.97 internal, 0.96 external
**Cardiac: amyloidosis screening**	Ultromics EchoGo Core + EchoGo Amyloidosis (Ultromics)	FDA 510(k) EchoGo Core (2019); EchoGo Amyloidosis K240860 (Nov 2024, first TAP-pathway clearance)	LV quantification and strain (Core); cardiac amyloidosis risk detection (Amyloidosis)	Multiple Ultromics-sponsored external-validation studies; TAP-pathway FDA review
**Cardiac: valvular CADx**	Echo IQ EchoSolv AS (Echo IQ); AISAP CARDIO (AISAP)	FDA 510(k) EchoSolv K241245 (2024); AISAP 510(k) (Aug 2024)	Severe aortic stenosis decision support (EchoSolv); multi-valve CADx (AISAP)	Vendor validation; AISAP first multi-module valvular AI clearance
**POCUS handheld cardiac**	DiA LVivo suite (DiA Imaging Analysis)	FDA 510(k) K210053, K222970, K243862 (2021-2025); CE mark	Automated LV EF, image-quality scoring, RV, PLAX/SAX, bladder volume	Multiple peer-reviewed validations of LVivo EF across handheld and cart systems
**POCUS multi-organ AI suite**	Exo Iris (Exo)	FDA 510(k); 14 AI apps as of June 2025	Multiorgan handheld AI suite (cardiac, lung, bladder, hip, thyroid, pleural effusion, consolidation)	Vendor validation studies; breadth exceeds single-domain competitors
**POCUS handheld (lung, bladder, GA)**	Butterfly Network: Auto B-lines, Auto Bladder Volume, GA Tool	Separate FDA 510(k) clearances per module (2020-2026)	B-line quantification from 6-s clips; automated bladder volume; blind-sweep GA	Vendor studies; GA Tool is the first blind-sweep GA clearance
**Regional anesthesia**	ScanNav Anatomy PNB (Intelligent Ultrasound)	FDA De Novo DEN220024 (Oct 2022; new device classification); CE mark	Real-time colour overlay of nerve-block anatomy across 10 peripheral-block regions	Bowness 2023 *Br J Anaesth*: 93.5% overall accuracy on nerve-block structure identification
**Vascular (Doppler)**	See-Mode AVA (See-Mode Technologies)	FDA 510(k) (Oct 2020); CE mark	Automated carotid and lower-extremity vascular analysis with structured reporting	Vendor validation; AVA is the first fully automated AI vascular-analysis platform cleared in the United States
**Interventional (liver ablation)**	BioTraceIO Lite (Techsomed)	FDA De Novo (Dec 2023, first ultrasound-based software for tissue-response prediction in liver ablation)	AI tissue-response prediction and margin guidance for thermal liver ablation	Techsomed vendor data; first-in-class clearance

K-numbers included where publicly verified.

These 3 categories are not a taxonomy to memorize; they matter because the pathway contrast in the rest of this article bends them in different directions. Radiology ultrasound AI tends to sit near segmentation and quantitative measurement, because high-quality images and expert operators make the harder task tractable and the payoff in measurement consistency is large. POCUS AI tends to sit near classification and lightweight detection, because images are lower quality and the operator needs near-instant feedback. Educational AI draws on all 3 but is judged not by diagnostic agreement but by improvement in learner competency (Figure 1). Figure 1B summarizes the clinical applications most mature at each pathway-by-task intersection.

**Figure 1:**
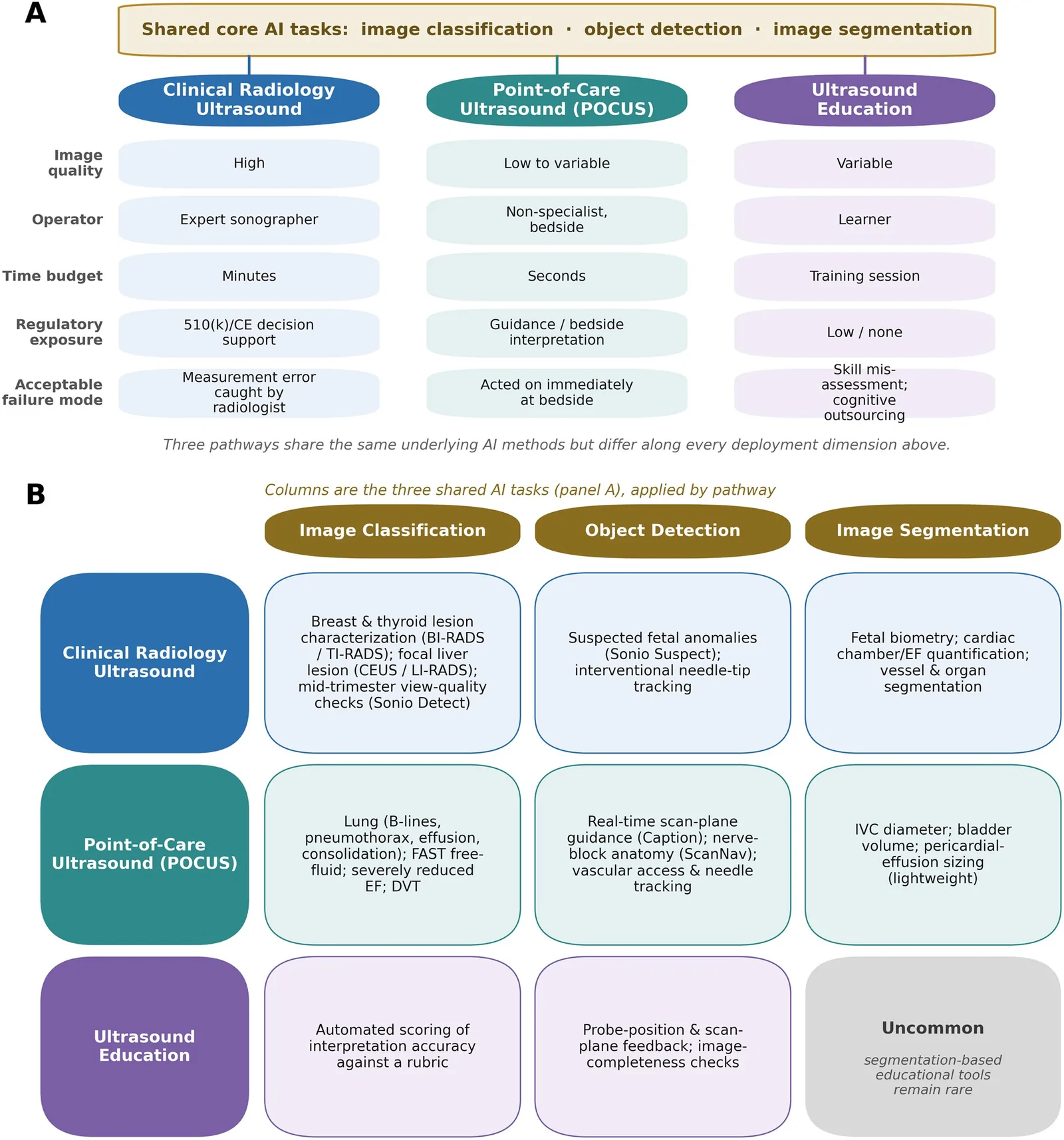
(A) The 3-pathway framework for ultrasound AI: clinical radiology ultrasound, the point-of-care bedside, and the ultrasound training program share core AI tasks but differ in image quality, operator skill, time budget, regulatory exposure, and acceptable failure modes. (B) Representative clinical applications by pathway (rows) and AI task category (columns); each cell lists the applications most mature at that intersection. The Education × Segmentation cell is grayed because segmentation-based educational tools remain uncommon.

Computer-aided detection in ultrasound predates the deep-learning era. Rule-based and classical machine-learning tools for breast and liver imaging were under academic and commercial development from the late 1990s onward, and their results shaped the expectations that modern deep learning later inherited. What changed in the last decade was the arrival of convolutional deep learning, catalyzed by the 2012 ImageNet and AlexNet results and the parallel rise of high-throughput graphics processing units (GPUs).^7^ It was not long before researchers found that transfer learning from natural-image datasets yields smaller benefits for medical imaging than commonly assumed, and that simpler models can match the performance of ImageNet-pretrained architectures.^8^ Deep networks also imported a genuinely new failure mode: adversarial vulnerability. Small, deliberately constructed perturbations, invisible to a human reader, can flip a classifier’s output, and medical-imaging pipelines have been shown to be attackable in exactly this way, a reminder that deep learning’s strengths and its fragilities arrived together.^9^ Natural-image networks learn from richly colored, object-dense scenes; ultrasound images are grayscale, speckle-dominated, low-contrast cross-sections of soft tissue in which the signal-to-noise ratio is low and the anatomy depends on probe position.^10^ The training-data abundance that made natural-image AI tractable is also largely absent, especially in POCUS, where image quality varies greatly by device, by operator skill, and by patient habitus within a single scan. Cross-device performance degradation is a recurring finding: models trained on one vendor’s cart-based images frequently drop several percentage points in area under the curve (AUC) when deployed on handheld devices or on images from a different manufacturer, a problem developed further in the cross-cutting challenges section below.^11^ Most of the failures catalogued later in this manuscript, generalization cliffs, cross-device fragility, label scarcity, are downstream effects of this mismatch.

## AI in radiology ultrasound

Upon first glance, radiology ultrasound imaging and POCUS appear to be worlds apart, especially to practitioners in each category who do not intersect regularly with colleagues in the other. Radiology-based ultrasound might be viewed by POCUS users as having amply supplied funding, the best equipment, image-interpretation expertise, and image-acquisition excellence, with little need for AI. In reality, clinical radiology ultrasound is under mounting strain: imaging demand has grown faster than the supply of sonographers and radiologists, interpretation backlogs lengthen, and burnout in both populations is well documented.^2^ Within this setting AI is not a luxury but a workforce multiplier, automating measurement, flagging findings, drafting structured reports, and reducing inter-reader variability, while leaving the expert hand movements of the sonographer and the final sign-off of the radiologist intact. Table 1 summarizes representative FDA-cleared and CE-marked AI-enabled ultrasound devices across the radiology and POCUS domains discussed in this and the next section.

### Breast and thyroid lesion characterization

Computer-aided detection for breast ultrasound predates the deep-learning era, but its clinical impact rose sharply with modern algorithms. In a 2025 prospective single-center study of 226 lesions in 222 patients, Irmici et al^12^ showed that a commercial AI decision-support system (Koios DS Breast) improved the diagnostic performance of radiologists at every experience level, with the largest gain seen in the junior breast radiologist. Parallel work in thyroid imaging has shown a complementary pattern. In 649 nodules from 347 patients at Duke University, Ashton et al. reported that the Koios DS Thyroid algorithm and radiologists achieved similar standalone performance (AUC 0.70 for both). The algorithm was more specific and the radiologist more sensitive; the best performance, and the most clinically useful one, came from combining the 2 (combined AUC 0.76), which reduced fine-needle-aspiration recommendations by 17% without loss of sensitivity, with ground truth anchored in surgical pathology, fine-needle aspiration, or 3-year follow-up.^13^ Both systems are FDA-cleared and CE-marked as decision support, and both sit squarely within the BI-RADS and TI-RADS reporting frameworks that radiologists already use. The generalizable lesson is that the largest absolute gains accrue to less-experienced readers; the more experienced reader gains less but still meaningfully, a deployment pattern worth naming explicitly rather than claiming uniform benefit.

### Obstetric ultrasound

Obstetric imaging is one of the most consolidated commercial AI footprints in clinical radiology ultrasound. Standard fetal biometry, biparietal diameter, head circumference, abdominal circumference, femur length, is highly protocolized and well suited to measurement automation. Every major cart-based vendor offers an automated biometry package, and validation studies show good inter-operator agreement and high completion rates, though automated estimates can slightly overestimate fetal weight and become less reliable in the third trimester.^14^ Beyond biometry, mid-trimester anomaly screening is the domain in which detection AI is most actively maturing; Sonio Detect provides real-time image-quality and view-completeness checks during the anatomy survey, while Sonio Suspect adds anomaly-detection decision support, both FDA-cleared and CE-marked to reduce missed structural anomalies.^15^

### Cardiac quantification

Cardiac ultrasound is the radiology-adjacent domain in which AI has moved most convincingly from research into routine reporting. Automated left-ventricular volumes, ejection fraction (EF), strain, and chamber quantification are now offered by every major vendor. The landmark demonstration was EchoNet-Dynamic, which performed beat-to-beat assessment of cardiac function with an LV-segmentation Dice similarity coefficient of 0.92, an EF mean absolute error of 4.1%, and heart-failure classification AUC of 0.97; on external validation the model preserved an EF mean absolute error of 6.0% and AUC of 0.96.^16^ Commercial expressions span Us2.ai, Ultromics EchoGo, Echo IQ, and vendor-native quantification packages. Echocardiography is formally cardiology-owned, but its interface with radiology ultrasound is dense and growing.

### Hepatic, vascular, and interventional imaging

Liver ultrasound AI has matured along 2 tracks. Quantitative steatosis assessment, the ultrasound analog of MRI-based hepatic fat quantification, now has academic and early commercial implementations in which quantitative ultrasound methods estimate hepatic fat fraction with clinically useful agreement against MRI-PDFF reference, reducing reliance on cross-sectional imaging for metabolic-dysfunction-associated steatotic liver disease surveillance.^17^ Elastography for fibrosis staging, long available on cart systems, is increasingly augmented with AI, including point-of-care implementations that identify hepatic fibrosis and steatosis, although published validation remains limited.^18^ For focal hepatic lesions, contrast-enhanced ultrasound (CEUS) AI aligned with the LI-RADS framework is an emerging research area, offering a radiation-free alternative to multiphase computed tomography in surveillance populations, though commercial penetration is minimal. In vascular imaging, early Doppler-AI work targets carotid stenosis grading, peripheral arterial disease characterization, and venous-duplex deep-vein thrombosis detection; however, commercial penetration lags the breast and obstetric domains by several years. AI-assisted needle-tip tracking, vessel-wall segmentation, and real-time plane prediction are appearing in selected cart-based and handheld interventional platforms, with the clearest published evidence so far in robotic femoral vascular access.^19^

### Reporting and workflow

Once an ultrasound study has been completed, the clock is still ticking until a report is finalized and uploaded into the electronic record. AI tools that assemble, structure, or draft reports have therefore been an early target of developer attention. Mehdiratta et al^20^ described an approach in which AI results are integrated into radiology reports using common data elements and DICOM structured reporting, demonstrating feasibility for quantitative cross-sectional findings; the same framework is directly applicable to ultrasound measurements such as biometry, EF, and organ volumes. Upstream of reporting, workflow AI anticipates what the operator wishes to do next, compiles saved images, and pre-places calipers, compressing the steps outside of transducer movement and scan-plane acquisition. These gains are modest per examination but compound across a busy department.

## AI in point-of-care ultrasound

POCUS has traditionally been seen, from the radiology perspective, as the more fragmented cousin of clinical radiology ultrasound, with small numbers of top-echelon researchers and educators pushing the field forward and differentiating it into its many specialty-driven iterations. Unlike their radiology colleagues, broader POCUS providers have only in the past decade begun to standardize their protocols and scanning approaches, and the label “POCUS” itself conceals significant heterogeneity. Emergency medicine uses ultrasound for trauma (FAST and extended FAST), shock differentiation, and procedural guidance among others. Critical care relies on POCUS for hemodynamic assessment, fluid-responsiveness prediction, and ventilator weaning. Anesthesiology uses it for regional nerve blocks and vascular access. Primary care increasingly uses it to screen abdominal, pelvic, and thyroid structures, and prehospital and austere-environment providers use it to triage.^21^ Each of these sub-settings has its own dominant pathologies, time pressures, and operator-skill distributions, and each pulls AI development in a different direction.

Accordingly, POCUS AI addresses a different problem than radiology ultrasound AI. The user is often not a dedicated imaging specialist, the device is frequently a handheld or compact cart with image quality far below the radiology baseline, the time budget can be measured in seconds, and the interpretation is typically completed by the scanning clinician in real time rather than passed to a downstream reader. POCUS AI is therefore built less around marginal accuracy gains in interpretation and more around enabling the scan to happen at all, and ensuring that what was seen is documented.

### Real-time scan-plane guidance

The clearest POCUS success is acquisition guidance. Narang et al^22^ showed in a 2-center prospective study that 8 nurses with no prior echocardiography experience, guided by the Caption Guidance deep-learning algorithm on a portable ultrasound system, acquired diagnostic-quality limited transthoracic echocardiograms in 98.8% of left-ventricular assessments, 92.5% of right-ventricular assessments, and 98.8% of pericardial-effusion assessments across 240 patients, a result that reshapes the workforce question in emergency and critical care. Comparable guidance systems exist for FAST, lung ultrasound, and vascular access. A parallel trajectory has appeared in regional anesthesia: ScanNav Anatomy (Intelligent Ultrasound), FDA-cleared in 2022, provides real-time color-overlay identification of nerve-block anatomical structures and has been externally validated with overall accuracy of 93.5% across peripheral nerve-block regions.^23^

### Automated interpretation at the bedside

Once a diagnostic image has been acquired, POCUS AI takes over interpretation tasks that would otherwise require formal training. Lung ultrasound AI has moved beyond B-line counting for pulmonary edema to pneumothorax screening (detecting absence of lung sliding), pleural-effusion quantification, and consolidation identification in pneumonia and acute respiratory distress syndrome. Inferior vena cava (IVC) diameter measurement for volume status, bladder-volume estimation, pericardial-effusion flagging, EF approximation, free-fluid detection in FAST, and compression-ultrasound AI for deep-vein thrombosis have all been reported in research and commercial settings.^21^ The interpretation task is narrower and more focused than in radiology: these tools answer focused questions (“is there free fluid?” “is the ejection fraction severely reduced?”) rather than produce a comprehensive finalized report. Calibrated uncertainty, developed further under Emerging methods, matters more in this pathway than in any other, because these outputs are consumed in real time by clinicians who may lack the experience to disagree with them. An AI that reports its own confidence gives the non-expert user a principled reason to pause and escalate; without that calibration, the same output invites automation bias exactly where the safeguards against it are weakest.

### Procedural guidance

POCUS AI is increasingly embedded in real-time procedural workflows: central venous access (vessel identification, needle-tip tracking, confirmation of intraluminal position), thoracentesis and paracentesis (target-fluid localization, safe-needle-trajectory suggestion), and lumbar puncture (midline identification and interspace selection). Early autonomous-scanning work on femoral vascular access suggests these systems can perform well when the anatomical target, a large vein, a fluid pocket, or an interspinous space, is well characterized, although cross-device generalization was not tested.^19^

### Documentation, quality assurance, and tele-POCUS

A durable and underappreciated POCUS problem is that many examinations are never documented. Aziz et al^24^ demonstrated in a UK emergency department that only 15 of 173 resuscitation patients (8.7%) had any POCUS documentation at baseline, a rate that improved substantially with a structured proforma and education intervention. AI-assisted POCUS platforms increasingly automate image selection, pathology annotation, report drafting, and, in some products, billing-code generation, converting documentation from a recurring burden into a background task. In programs responsible for credentialing, these platforms also supply longitudinal competency data with little additional clinician effort. Tele-POCUS, remote AI- or expert-guided scanning, extends the same logic to settings where an on-site specialist is unavailable: rural emergency departments, prehospital transport, and humanitarian deployments. The infrastructure behind tele-POCUS deserves more scrutiny than it usually receives. Real-time remote guidance requires a sustained video uplink of several megabits per second and round-trip latency low enough, roughly under 200 ms, for probe-adjustment instructions to feel synchronous; store-and-forward quality assurance tolerates delay but still requires DICOM routing, de-identification, and archive integration that many POCUS programs lack. Where the AI inference runs matters as much: on-device (edge) inference avoids the network entirely but is constrained by handheld compute and battery, while cloud inference inherits every connectivity, latency, and security constraint above. In rural and austere settings, precisely where tele-POCUS promises the most, these prerequisites are least likely to be met, and pilot-study feasibility should not be mistaken for deployment readiness.

## AI in ultrasound training and education

The third setting for ultrasound AI is that of ultrasound education, focused mainly on medical schools and broadly applicable to residency education as well. The needs of these learners invariably include a shortage of instructors, a shortage of structured curricular material, and limited exposure to scanning opportunities. Unlike in the clinical settings above, the goal is not to complete an examination but to build the cognitive and psychomotor skills that make examinations possible at all.

Educational AI therefore sits apart from clinical AI in ways that are easy to overlook. An AI that simply performs the interpretation deprives the learner of the very feedback loop that builds skill, the unique design constraint of educational AI is to teach, not to do, and uncritical deployment risks the very cognitive outsourcing that medical-education literature has repeatedly flagged.^25^ AI-guided scan training systems show the learner in real time whether a given probe position produces a diagnostic image, and provide structured feedback on probe orientation, depth, and gain. Competency-assessment AI builds on existing POCUS competency frameworks to augment direct instructor observation with an objective, reproducible score that supports credentialing and longitudinal tracking; emerging examples grade learners on image completeness, probe-manipulation efficiency, and interpretation accuracy against a standardized rubric, producing a trajectory that program directors can audit rather than infer from case-log counts.^26^ Simulation platforms coupled to AI offer pathologies at a frequency unseen in most clinical rotations. Ready availability of practice scans including pathologies such as severe aortic stenosis, tamponade, pediatric cardiac views, and others reduce dependence on patient encounters for exposure to rare conditions. Finally, AI democratizes access to expertise: a learner in a low-resource setting can receive the same real-time feedback as a learner in an academic medical center, which is among the strongest ethical arguments for the technology.

A caveat worth stating explicitly is skill atrophy. If AI performs the interpretation throughout training, the trainee may never build the judgment that AI is supposed to support. Educational AI design must therefore resist the temptation simply to interpret, and instead scaffold the learner toward independent interpretation, a constraint absent from the clinical pathways above.

## Cross-cutting challenges: what every deployment must navigate

Several challenges span all 3 pathways but are weighted differently in each. They are grouped here because the underlying issues are shared; the deployment response is not.

### Training data is asymmetric

Radiology ultrasound has relatively large, homogeneous datasets from cart-based vendors, but they over-represent tertiary-care populations. POCUS suffers from device diversity (Butterfly, Kosmos, GE Vscan, Philips Lumify, Mindray, EDAN, Clarius, Sonosite, and more), protocol variation across specialties, and label scarcity; most POCUS scans are not annotated. Educational AI has the smallest and most heterogeneous training pool of the 3. Pediatric ultrasound AI is a pronounced further gap: Published work is concentrated in developmental dysplasia of the hip and pediatric appendicitis, with more limited evidence for other conditions such as hypertrophic pyloric stenosis, and reported performance is largely single-center with limited external validation. Consent requirements for pediatric data use further constrain the multicenter pooling that adult applications have benefited from.^27^ High-body-mass-index patients, pediatric hearts, and non-white surface-landmark appearances are underrepresented across the field. Methods that reduce dependence on manual labels (self-supervised pretraining, unsupervised domain adaptation, and foundation-model transfer) are discussed under Emerging methods.

### Public datasets and the anatomy of an ideal one

A growing number of open datasets partially offset this asymmetry and now anchor much of the published benchmarking. In cardiac imaging, the Stanford Center for Artificial Intelligence in Medicine and Imaging (AIMI) collections are the de facto standard: EchoNet-Dynamic provides more than 10 000 apical-4-chamber echocardiographic videos with expert tracings and ejection fractions,^16^ and EchoNet-LVH extends the approach to parasternal-long-axis views for left-ventricular-hypertrophy phenotyping.^28^ Widely used resources in other domains include the Breast Ultrasound Images dataset (BUSI, 780 annotated images),^29^ carotid-artery and annotated liver ultrasound collections hosted on Kaggle, lung ultrasound image sets for automated disease classification on Mendeley Data, and the Stanford AIMI thyroid cine-clip dataset. These resources have democratized method development, but they share structural weaknesses: most are single-center and single-vendor, modest in size by natural-image standards, built from still frames rather than cine loops, sparsely documented with respect to device, preset, and patient demographics, and of heterogenous annotation quality. An ideal ultrasound AI dataset, the yardstick against which both public and proprietary collections should be judged, would be multicenter and multivendor including handheld devices; demographically representative and reported (age, sex, body-mass index, skin tone); cine-loop based, since ultrasound is a temporal modality; annotated by multiple experts with documented inter-reader variability; accompanied by acquisition metadata (system, transducer, preset); published with fixed train/test partitions and version control; and distributed under clear licensing with a mechanism for reporting labeling errors.

### Cross-device generalization failure

A model trained on one vendor’s images often degrades sharply on another’s and degrades further on handheld devices with different beamforming and gain behavior.^11^ The pattern is not unique to ultrasound; in a now-canonical chest-radiograph study, deep-learning pneumonia classifiers transferred poorly across institutions, driven largely by subtle dataset-level artifacts rather than physiological differences.^30^ The same mechanism operates in ultrasound, different vendors produce systematically different speckle statistics, beamforming, and on-device processing, and is the reason generalization across devices should be a primary validation endpoint rather than a secondary one. Figure 2 contrasts how this drift may unfold in clinical radiology ultrasound versus POCUS and illustrates how post-deployment monitoring followed by a successfully validated vendor update may recover performance toward baseline after drift is detected.

**Figure 2:**
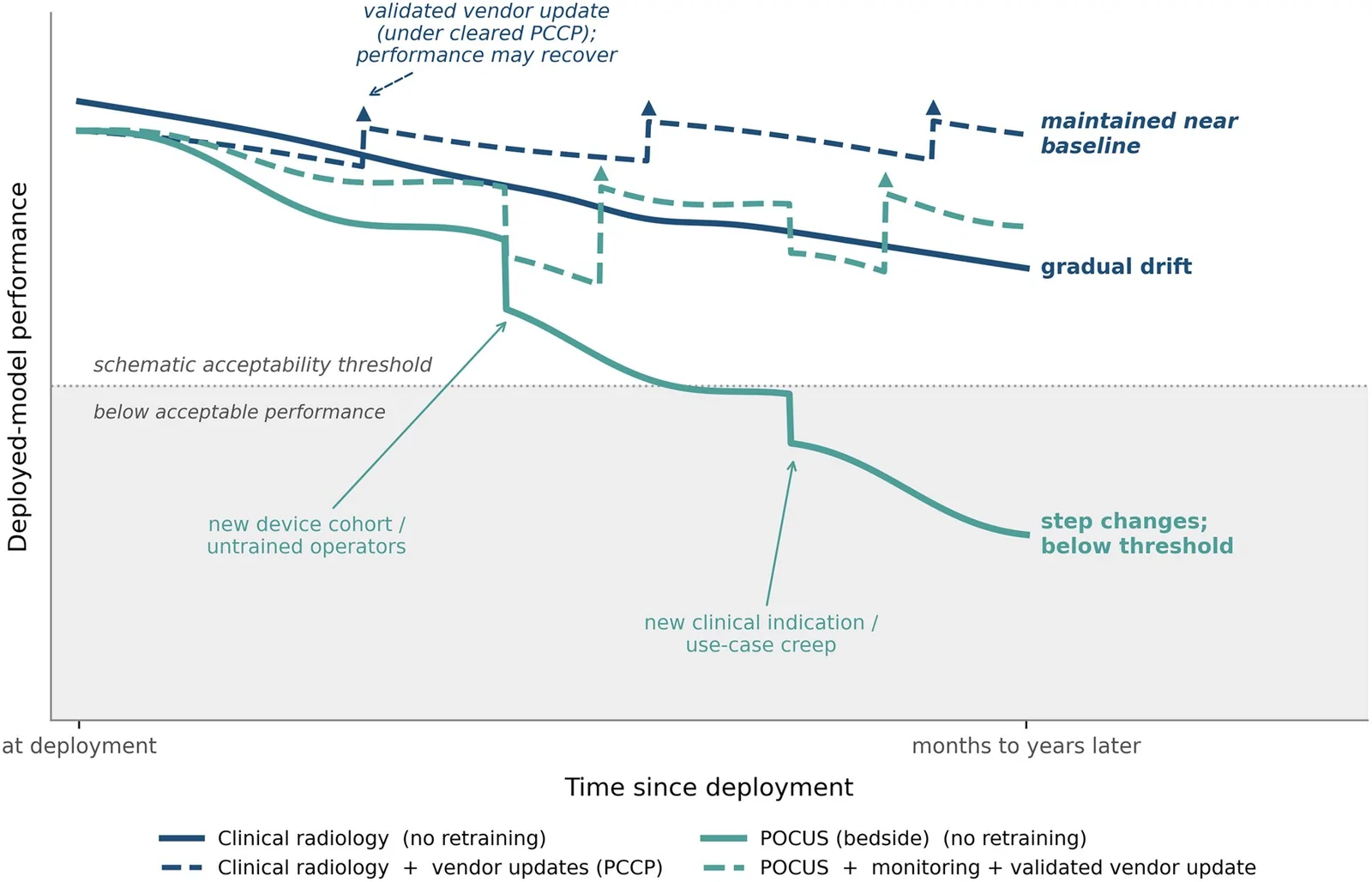
Schematic contrast of deployed-model performance drift over time between clinical radiology ultrasound and POCUS, with and without continuous improvement. Solid curves show the no-retraining trajectory: radiology drifts slowly, while POCUS degrades faster with step changes at new device cohorts and new indications, eventually dropping below an acceptability threshold. Dashed curves show the same pathways with scheduled vendor updates issued under a Predetermined Change Control Plan (PCCP) (radiology) or post-deployment monitoring plus validated vendor updates (POCUS); triangle markers denote update or revalidation events. After a successfully validated update, performance may recover toward its original level. Axes are illustrative and carry no numeric values; the figure is conceptual.

### Clinical validation and reporting standards

The AI-specific reporting frameworks, CLAIM for medical-imaging AI studies, SPIRIT-AI and CONSORT-AI for trial protocols and completed trials, DECIDE-AI for early-stage clinical evaluation, and TRIPOD + AI for prediction models, provide a shared vocabulary for ultrasound-AI work, but adherence remains uneven.^31–35^ A substantial fraction of published ultrasound AI performance claims come from single-center retrospective datasets without external validation, and the gap between published performance and deployed performance remains large (Table 2). Table 3 summarizes the key studies cited in this review with their validation approach and principal limitations; read column-wise, it makes the maturity gradient explicit: Cardiac quantification and breast decision support rest on prospective or externally validated evidence, much of POCUS interpretation rests on single-center or feasibility-grade evidence, and educational AI has barely begun to accumulate controlled evidence at all.

**Table 2:** Reporting, validation, and development-practice standards for medical AI applicable to ultrasound, chronological order.

Standard	Type	Purpose	Stage/activity addressed	Reference
**CLAIM**	Reporting checklist	Medical-imaging AI studies	Model reporting	Mongan et al. *Radiol Artif Intell*. 2020;2:e200029
**SPIRIT-AI**	Reporting checklist	Clinical trial protocols involving AI interventions	Trial design	Rivera et al. *Lancet Digit Health*. 2020;2:e549–e560
**CONSORT-AI**	Reporting checklist	Completed clinical trials of AI interventions	Trial reporting	Liu et al. *Lancet Digit Health*. 2020;2:e537-e548
**GMLP Guiding Principles**	Development-practice framework	10 joint FDA/MHRA/Health Canada principles for ML/AI medical device development	Whole lifecycle (data, training, validation, monitoring)	FDA/MHRA/Health Canada joint publication, October 2021
**DECIDE-AI**	Reporting checklist	Early-stage clinical evaluation of AI decision support	Early-stage clinical evaluation	Vasey et al. *BMJ*. 2022;377:e070904
**NICE Evidence Standards Framework for Digital Health Technologies**	Development-practice/procurement framework	UK NHS tiered evaluation framework for digital health, covering AI/ML	Procurement and commissioning	NICE ESF, updated August 2022
**TRIPOD + AI**	Reporting checklist	Clinical prediction models using ML or regression	Prediction-model reporting	Collins et al. *BMJ*. 2024;385:e078378

**Table 3:** Summary of key studies cited in this review: pathway, AI task, study design and cohort, reported performance, validation approach, and principal limitations

Study (ref)	Pathway/task	Design and cohort	Reported performance	Validation	Key limitations/readiness
**Irmici 2025^12^**	Radiology: breast lesion classification	Prospective, single tertiary center; 226 lesions/222 patients	Improved diagnostic performance at every reader-experience level; largest gain for the junior breast radiologist	Internal, prospective	Single center; one commercial system; FDA-cleared/CE-marked decision support
**Ashton 2024^13^**	Radiology: thyroid risk stratification	Retrospective, single center; 649 nodules/347 patients	AUC 0.70 (AI) vs 0.70 (radiologists); combined AUC 0.76; −17% FNA recommendations, no sensitivity loss	Internal; pathology/follow-up ground truth	Modest standalone accuracy; benefit is in combination, not replacement
**Ambroise Grandjean 2025^14^**	Radiology: fetal biometry (segmentation)	Pilot feasibility study	Good inter-operator agreement and high completion; slight fetal-weight overestimation, weaker in the third trimester	Internal, pilot	Pilot scale; feasibility-grade evidence
**EchoNet-Dynamic, Ouyang 2020^16^**	Cardiac: LV segmentation/EF	Retrospective; 10 030 videos	Dice 0.92; EF MAE 4.1%; heart-failure AUC 0.97	External (EF MAE 6.0%, AUC 0.96)	Retrospective; cart-based apical-4-chamber; research model
**Caption Guidance, Narang 2021^22^**	POCUS: real-time acquisition guidance	Prospective, 2 centers; 8 novice nurses; 240 patients	Diagnostic-quality limited TTE in 98.8% (LV), 92.5% (RV), 98.8% (pericardial effusion)	Prospective, multicenter	Acquisition only; interpretation still expert-dependent
**ScanNav Anatomy, Bowness 2023^23^**	POCUS: nerve-block anatomy detection	External validation study	Overall accuracy 93.5% across peripheral nerve-block regions	External	Identification aid, not diagnosis; downstream decisions expert-dependent
**AI-GUIDE, Brattain 2021^19^**	POCUS: guided femoral venous access	Porcine model	Catheter insertion ∼80 s; 1.1-1.3 needle attempts (comparable to published figures for experienced operators)	Preclinical	Animal model; well-characterized large-vessel target; generalization untested
**Pokaprakarn 2022^51^**	POCUS: GA from blind sweeps	Low-resource-setting study	GA estimation from blind sweeps by minimally trained users	Held-out test set; no truly external cohort	Research prototype (validated in North Carolina and Zambia); broader external and real-world validation still limited
**EchoCLIP, Christensen 2024^48^**	Emerging: vision-language multi-task echo	Retrospective; >1M video-report pairs	EF MAE 7.1% (external); device-detection AUC 0.84-0.97	External	Research-only; no clearance; broad but shallow task coverage
**UltraSam, Meyer 2026^47^**	Emerging: ultrasound segmentation foundation model	280 000+ images, 43 datasets	Improved adaptation vs natural-image backbones	Benchmark-level	No clinical validation; research artefact
**Han 2020^17^**	Radiology: quantitative liver steatosis	Single-center quantitative-US study	Clinically useful agreement with MRI-PDFF reference	Internal	Single center; early commercial maturity

Abbreviations: AUC = area under the curve; CI = confidence interval; DVT = deep-vein thrombosis; EF = ejection fraction; FNA = fine-needle aspiration; GA = gestational age; LV = left ventricle; MAE = mean absolute error; TTE = transthoracic echocardiogram.

### Regulation across jurisdictions

In the United States, most AI-enabled ultrasound devices are cleared via 510(k), with a smaller number via De Novo; the FDA’s final guidance on Predetermined Change Control Plans, issued in December 2024 and updated in August 2025, now governs how adaptive algorithms may be updated without a new marketing submission.^36^ In the European Union, devices fall under the Medical Device Regulation supplemented by the AI Act, which imposes additional obligations on high-risk AI systems. In the United Kingdom, the Medicines and Healthcare products Regulatory Agency (MHRA) operates the Software and AI as a Medical Device Change Programme; CE-marked devices remain accepted in Great Britain until 30 June 2028 for legacy MDD/AIMDD devices and until June 30, 2030 for EU MDR/IVDR devices, and the MHRA’s AI Airlock pilot (announced April 2024, commenced May 2024) provides a regulatory-sandbox pathway for novel AI devices.^37^ The 2021 Good Machine Learning Practice principles jointly issued by the FDA, MHRA, and Health Canada now anchor lifecycle-management expectations across the 3 regulators (Table 4).^38^

**Table 4:** Regulatory pathways for AI-enabled ultrasound devices across 5 jurisdictions and the international harmonization body.

Jurisdiction (regulator)	Primary pathway	Adaptive AI algorithms	Key milestones (with dates)	Reference
**United States (FDA)**	510(k) for most AI-enabled ultrasound devices; De Novo for novel indications (eg, Caption AI)	Predetermined Change Control Plan (PCCP); final guidance December 2024, updated August 2025	FDA AI/ML action plan (2021); GMLP joint principles (Oct 2021); PCCP final guidance (Dec 2024, updated Aug 2025)	FDA PCCP guidance, December 2024 (updated August 2025)
**European Union**	Medical Device Regulation (MDR 2017/745) supplemented by the AI Act for high-risk systems.	MDR change procedures; AI Act risk-based obligations and post-market monitoring	MDR applicable May 2021; AI Act entered force August 2024; prohibited-practices provisions applied February 2025; high-risk AI obligations deferred by the 2026 AI Omnibus to December 2027 (August 2028 for AI in regulated medical devices)	EU MDR 2017/745; EU AI Act (Regulation 2024/1689)
**United Kingdom (MHRA)**	Software and AI as a Medical Device Change Programme; CE marking accepted in GB until 30 June 2028 (legacy MDD/AIMDD) or 30 June 2030 (EU MDR/IVDR); UKCA route already available	AI Airlock regulatory sandbox (announced April 2024, commenced May 2024)	UK MDR post-Brexit framework; Change Programme roadmap (updated 14 June 2023); AI Airlock announced April 2024, commenced May 2024	MHRA SaMD/AI Change Programme roadmap, 14 June 2023
**China (NMPA)**	Class II/Class III medical device registration with AI-specific technical review	NMPA technical-review guidelines for AI-enabled medical devices (2019 onward)	Technical review guidelines for deep-learning AI medical devices published 2019; technical review guideline updated 2022	NMPA guidance for AI medical devices, 2019/2022
**Japan (PMDA)**	SaMD classification with AI/ML-specific technical review	IDATEN framework (Improvement Design within Approval for Timely Evaluation and Notice) for AI/ML change management, launched 2020	SaMD framework since 2014; IDATEN for AI updates (2020)	PMDA SaMD guidance; IDATEN framework
**International (IMDRF)**	Non-clearance harmonization body	Publishes guidance adopted by multiple national regulators	Software as a Medical Device framework (2013); Good Machine Learning Practice guiding principles (N88:2025)	IMDRF SaMD framework; IMDRF GMLP guiding principles (N88:2025)

### Lifecycle governance and auditability

Regulatory clearance is a single point-in-time event; safe clinical use is a lifecycle (Figure 3). Institutions deploying ultrasound AI increasingly need what regulators now expect of manufacturers: a named owner for each deployed model, an inventory of models and versions in clinical use, documented training-data provenance, performance-monitoring dashboards with predefined drift triggers, a defined process for adopting or declining the vendor model updates that manufacturers issue under an FDA-cleared Predetermined Change Control Plan, and for retiring a model from clinical use,^36^ and an audit trail recording which model version contributed to which examination. Institution-level scaffolding for this accountability now exists: the NIST AI Risk Management Framework and ISO/IEC 42001 define who governs, who measures, and who responds when a model degrades,^39^^,^^40^ and healthcare-specific translations of these frameworks are emerging from health-system coalitions. For the ultrasound department, the practical test of audit-readiness is the ability to answer, for any AI-touched examination, 4 questions: which model and version produced the output, what data it was trained and validated on, who is monitoring its current performance (and by what means), and what happens when that performance drifts. Without such structures, the liability exposure described below has no institutional answer, and accountability for a silent model failure lands by default on the clinician who used the tool.

**Figure 3:**
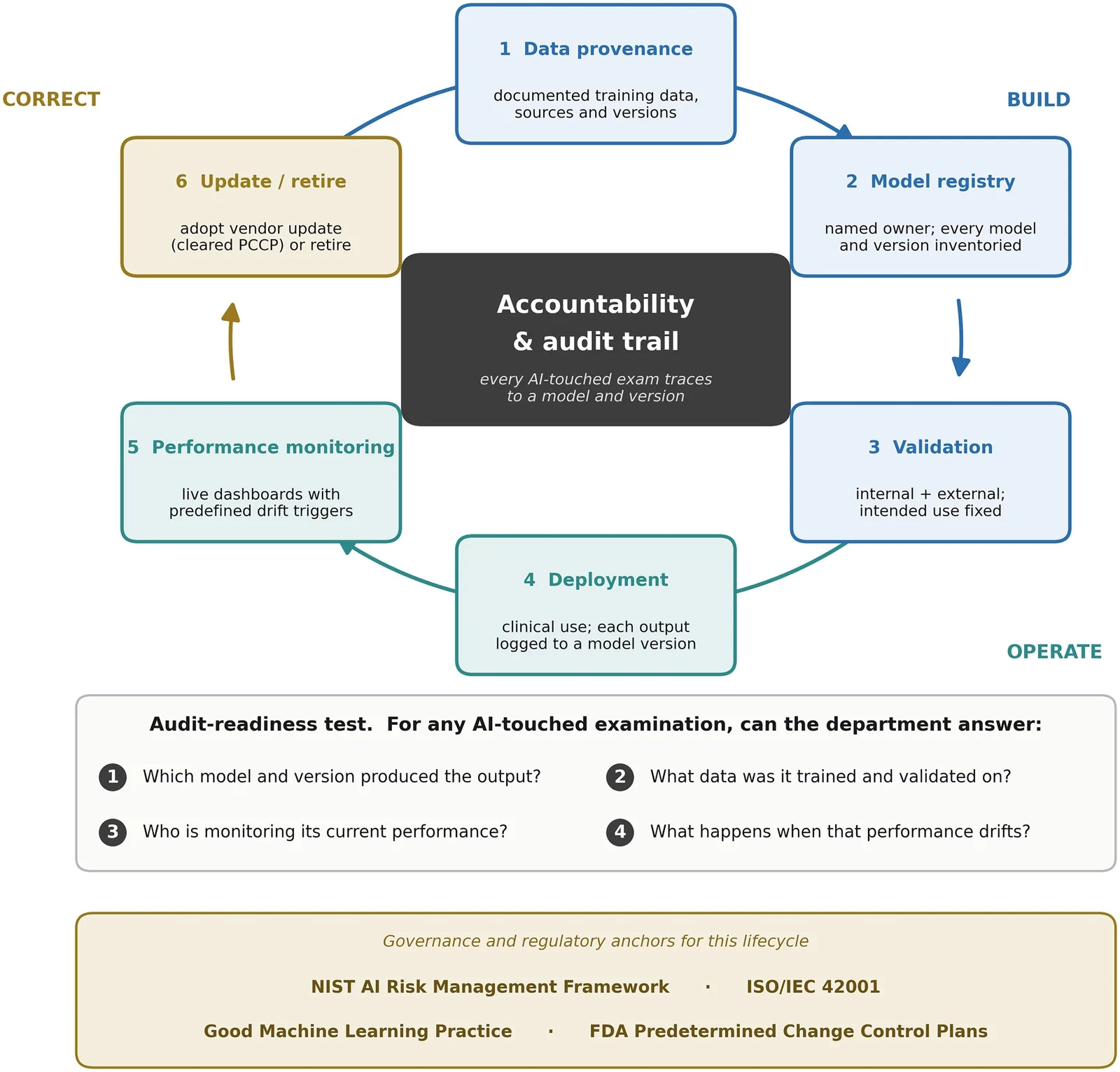
Institutional AI lifecycle governance and auditability. Safe clinical use of an AI tool is a continuous loop rather than a one-time clearance: data provenance and a model registry (named owner, version inventory) feed validation and deployment; deployed performance is monitored against predefined drift triggers; and detected drift prompts either a vendor model update (issued under the manufacturer’s cleared PCCP) or controlled retirement, with every stage logged to an auditable trail so that each AI-touched examination traces to a specific model and version. The 4-question audit-readiness test summarizes what a deploying department must be able to answer. The lifecycle is operationalized by the NIST AI Risk Management Framework, ISO/IEC 42001, Good Machine Learning Practice, and FDA Predetermined Change Control Plans.

### Cybersecurity of AI-enabled and handheld devices

A handheld ultrasound probe paired with a phone or tablet is an Internet-of-Things endpoint on a clinical network, and it inherits the vulnerabilities of this class: consumer-grade operating systems, cloud synchronization of patient images, intermittent patching, bring-your-own-device usage, and a physical portability that makes loss and theft routine. Networked AI inference widens the attack surface further, and modern convolutional networks carry a demonstrated susceptibility to adversarial image manipulation.^9^ Regulators have moved: in the United States, section 524B of the Federal Food, Drug, and Cosmetic Act now requires premarket cybersecurity documentation, a software bill of materials, and postmarket vulnerability management for connected devices.^41^ Institutional mitigations parallel those for other medical IoT: device inventories and network segmentation, encryption of images at rest and in transit, zero-trust access architectures, and anomaly detection on device traffic.^42^ None of this is exotic, but it is routinely bypassed when handheld devices are purchased at department level outside biomedical-engineering and information-security procurement, which is precisely how many POCUS programs have acquired them to date.

### Bias, equity, and patient-facing disclosure

Underrepresentation of high-body-mass-index patients, pediatric populations, and non-white skin tones in training data can translate directly into degraded real-world performance in those groups. Disclosure practices are immature: when an AI contributes materially to a reported finding, patients are rarely informed, and consent practices vary by institution. Emerging guidance from the UK Information Commissioner’s Office and the EU AI Act may, depending on the specific processing and legal trigger, call for some disclosure, and several US health systems have begun adding AI-use language to imaging consent. The practical argument for disclosure is that a patient who knows AI contributed to their scan is better positioned to ask informed questions, seek a second opinion, and report errors that would otherwise go unobserved; the legal argument is that an undisclosed AI contribution may, in some jurisdictions, bear on whether a patient’s consent was adequately informed.

### Reimbursement, liability, and environmental cost

Artificial intelligence-enabled ultrasound reimbursement in the United States remains patchwork. The American Medical Association’s Current Procedural Terminology Category III code 0932T (effective January 2025) covers AI-derived noninvasive detection of heart failure from echocardiographic video, but Category III codes do not guarantee payment and conversion to permanent Category I codes is slow and uneven.^43^ In the United Kingdom, NHS procurement has moved faster, funded in part by the AI in Health and Care Award. Liability remains unsettled: a recent legal analysis of liability risk from healthcare AI tools found that courts continue to hold individual clinicians to the existing standard of care, and physician reliance on an AI recommendation offers only limited protection when the outcome is poor.^44^ Environmental cost is also becoming part of the procurement calculus given the high energy uses potentially involved in foundational model training on large caches of GPUs.

## Emerging methods and near-term future

Several methodological and deployment trajectories are most likely to shape ultrasound AI over the next 3 to 5 years.

### Ultrasound foundation models

A foundation model is a large AI model trained once on a broad image collection and then adapted to multiple specific tasks, rather than built from scratch for each clinical question. Early examples in imaging began with Segment Anything, trained on natural images, followed by its medical-imaging adaptation MedSAM, fine-tuned on over 1.5 million medical image-mask pairs.^45^^,^^46^ An ultrasound-specific example, UltraSam, was trained on an open-access collection of more than 280 000 images spanning 43 datasets and 50-plus anatomical structures.^47^ The clinical promise is less engineering effort per new application and, because the underlying model has seen a much wider range of patients and devices than any single vendor’s dataset, potentially better performance on rare pathologies and underrepresented populations (Figure 4).

**Figure 4:**
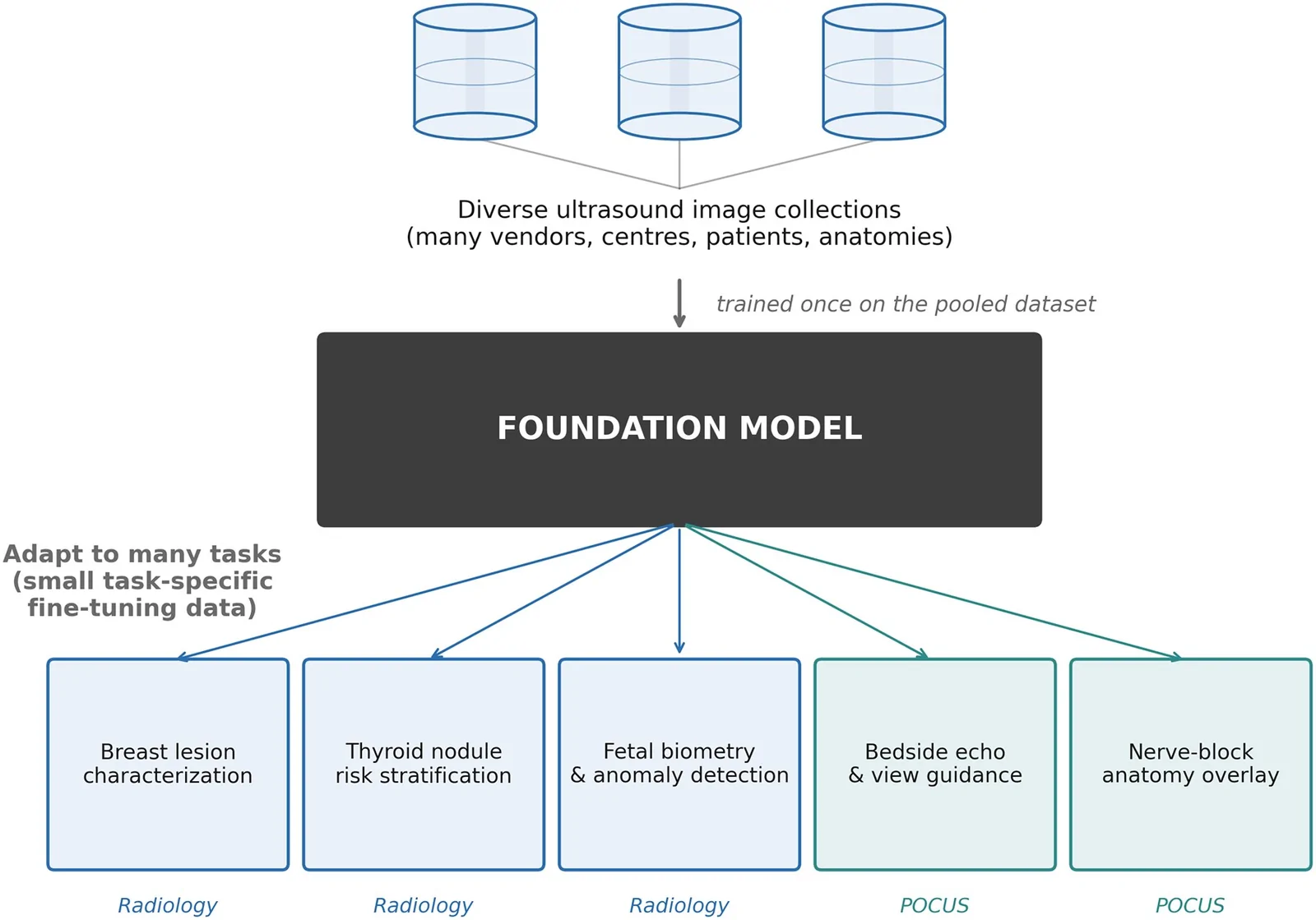
The foundation-model concept for ultrasound: a large pooled ultrasound image collection is used once to train a shared backbone, which is then adapted to multiple clinical tasks (breast, thyroid, obstetric, cardiac, POCUS) with small amounts of task-specific fine-tuning data.

### Vision-language and multivideo models

A second line of work trains a single AI model on paired ultrasound images and their clinical reports, so that the model learns both what a finding looks like and how it is described. EchoCLIP, trained on more than 1 million cardiac-ultrasound videos and the associated expert reports, can perform a range of cardiac tasks (estimating ejection fraction, flagging implanted devices) without being separately trained for each one, with an EF mean absolute error of 7.1% on external data and AUCs between 0.84 and 0.97 for device detection.^48^ A follow-on system, EchoPrime, extends this to multiple video inputs at once, closer to how a reader actually interprets a study. These approaches raise the natural question of whether an AI could 1 day orchestrate an entire ultrasound workflow, scan guidance, measurement, interpretation, and report drafting, as a set of cooperating agents. The answer is uncertain at this time, but the building blocks are converging.

### Self-supervised learning, federated learning, synthetic data, and uncertainty quantification

Self-supervised and unsupervised learning directly attack the label-scarcity and cross-device problems named earlier. Self-supervised pretraining learns image representations from unlabeled scans, of which every ultrasound department has an abundance, and has been shown in medical imaging to improve both label efficiency and out-of-distribution robustness.^49^ Unsupervised domain adaptation explicitly aligns feature distributions across vendors and devices, offering a remediation path for the cross-device degradation described earlier, and unsupervised anomaly-detection and clustering methods can surface candidate imaging biomarkers that no annotator was asked to label. The foundation models described above are the logical end point of this trajectory: pretrain once on broad, largely unlabeled collections so that each new clinical task needs only a small, labeled image set. Federated learning addresses the consent and privacy constraints that limit ultrasound data pooling, especially for pediatric and rare-disease applications. Synthetic-image generation using diffusion models increasingly supplements scarce training data and allows controlled studies of bias and failure modes. Explainability methods, saliency maps, attention overlays, prototype-based reasoning, are increasingly expected by regulators and users, but they remain a partial answer; the risk of over-trusting a convincing-looking post-hoc explanation is well documented. An adjacent and less-discussed need is calibrated uncertainty: a POCUS AI that reports “moderately-to-severely reduced ejection fraction” with a quantified confidence interval is a clinically actionable output, whereas the same model without that calibration is a liability risk.^50^

### Robotics coupled with ultrasound AI

A distinct direction pairs ultrasound AI with robotic actuators to automate invasive procedures. Fully autonomous scanning robots have been explored but remain research prototypes; the more immediate clinical application adds a small robotic mechanism to a handheld transducer to perform guided procedures such as biopsy or vessel cannulation. The AI-GUIDE device developed at MIT Lincoln Laboratory illustrates the approach: the handheld system analyses the real-time ultrasound image, directs the user to the correct transducer position through on-screen instructions rather than asking them to interpret the image itself, and then robotically advances a needle and guidewire into the femoral vein. In a porcine model, the device achieved catheter insertion in approximately 80 s with 1.1-1.3 needle attempts, performance comparable to previously reported figures for experienced operators performing human femoral access.^19^ Whether this framework generalizes from a well-characterized target such as a large vein to more subtle anatomy remains to be tested.

The near-term future of POCUS is on-device 3- and 4-dimensional imaging with on-edge AI, in which the clinician is responsible for transducer placement and the device is responsible for plane selection, measurement, interpretation, and documentation. Current handheld computing hardware is not yet at the latency and power envelope this workflow demands, but the direction is established. Supporting that direction, and tele-POCUS at scale, will require real-time data frameworks built for high-velocity streams: continuous ingestion, buffering, and event processing of cine data at rates that conventional PACS-centered imaging infrastructure was never designed to sustain. The strongest ethical case for ultrasound AI sits in global health: approximately two-thirds of the world’s population lacks reliable access to diagnostic imaging, and handheld ultrasound paired with AI is the only imaging modality with a credible path to universal coverage. Pilot deployments in low-resource settings, including sub-Saharan Africa, have demonstrated feasibility, but the evidence base is thin and the failure modes are understudied.^51^  Figure 5 locates the landmark methodological, clinical, commercial, and regulatory milestones that frame this section on a shared 2012-2025 timeline.

**Figure 5:**
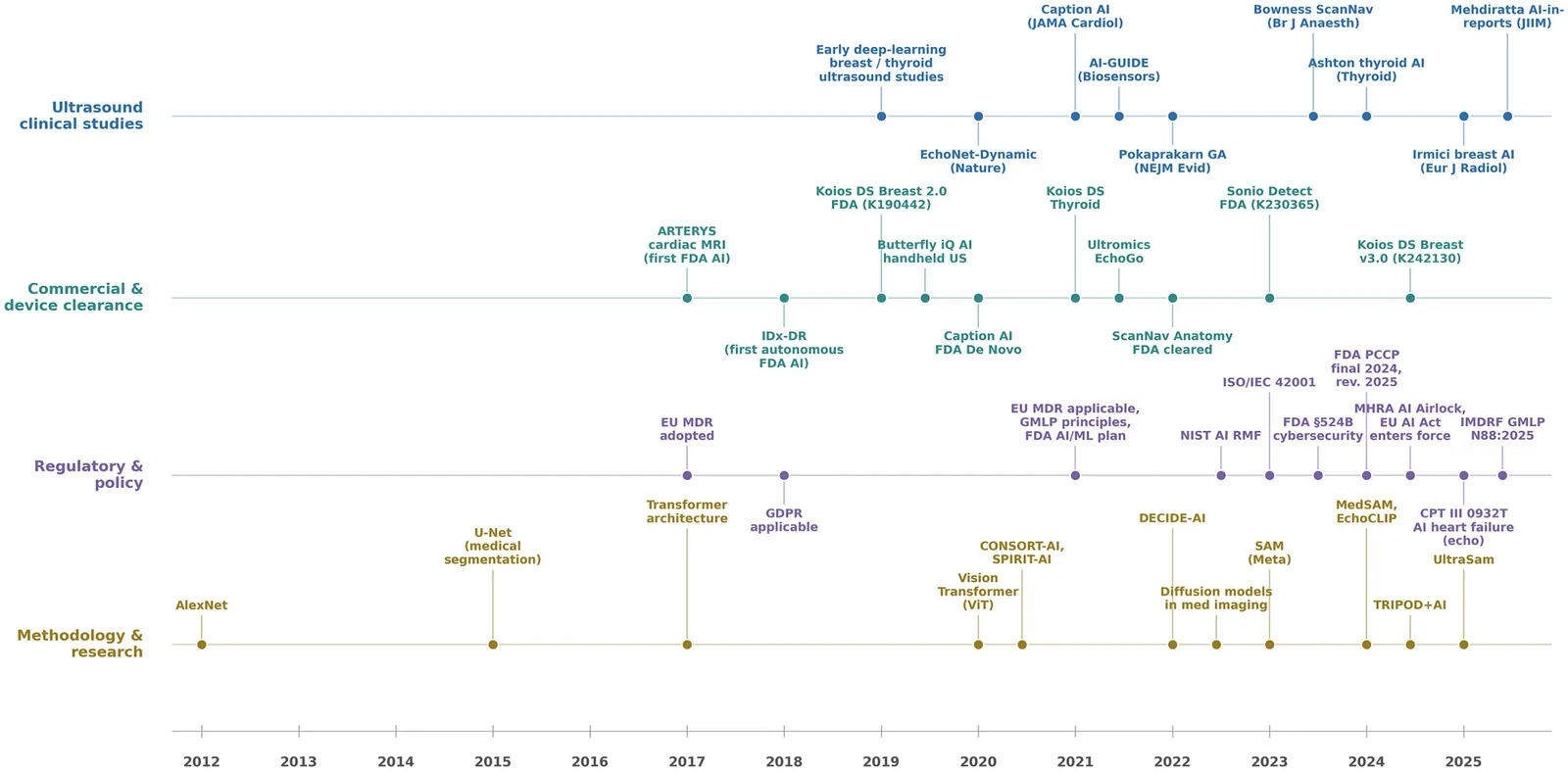
Milestones in ultrasound-relevant AI from 2012 to 2025, grouped into 4 lanes: ultrasound clinical studies, commercial and device clearance, regulatory and policy, and methodology and research. Detailed version with specific K-numbers, regulatory guidance dates, and methodology milestones.

## Conclusion and recommendations

Artificial intelligence in ultrasound is better described as 3 related fields than as one. Radiology ultrasound, point-of-care ultrasound, and ultrasound training share the same underlying algorithms and foundation models, but they differ in image quality, operator skill, time budget, regulatory exposure, and acceptable failure modes, and therefore in what AI can safely deliver today. A tool optimized for one pathway rarely translates to another without re-engineering, and treating the field as a monolith has been a costly and repeated error. The near-term question is not whether AI will reshape ultrasound; it already has. The more useful question is which pathway is ready for a given deployment, and that question is answered not by algorithmic sophistication but by honest attention to the deployment context and its constraints.

Several research priorities follow directly from the gaps catalogued above. The field needs prospective, multicenter validation of POCUS AI on handheld devices in the hands of real-world users, rather than archived cart-based images; public multi-vendor datasets with cine loops, demographic and device metadata, and fixed test partitions; pediatric-specific model development and validation; trials that measure patient outcomes rather than reader agreement; deployable uncertainty calibration, so that models report not just answers but confidence; auditable lifecycle governance that assigns institutional ownership of model monitoring, retraining, and retirement; and security architectures appropriate to AI-enabled mobile devices. Each of these maps to an unresolved challenge named in this review, and none is answered by better benchmark scores alone.

For the clinician entering ultrasound practice today, 3 practical points follow (Figure 6). First, the cleared indications for use and labeling, not the regulatory pathway alone, define the breadth of a tool’s validated claim: the pathway (510(k), De Novo, CE mark, UKCA) is only a loose proxy, as a 510(k) can authorize autonomous detection while a De Novo may cover only image-acquisition guidance. Second, performance reported from the center that developed a tool is rarely the performance the clinician will see in their own department, and external-validation data should be asked for and read carefully. Third, an AI that contributes materially to interpretation deserves acknowledgement in the report and, increasingly, in the patient conversation. These are habits worth forming now, before they become expectations.

**Figure 6:**
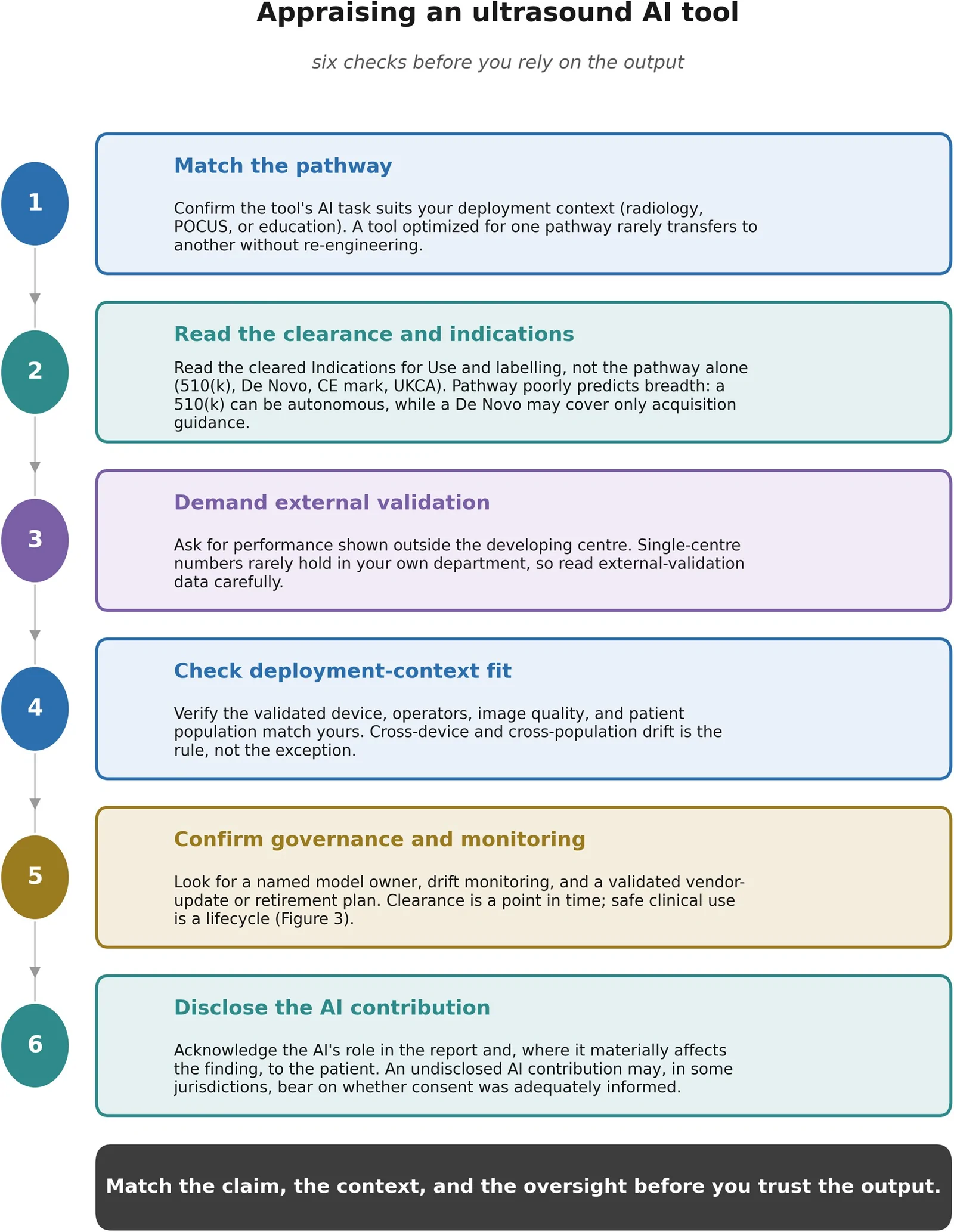
A practical checklist for appraising an ultrasound AI tool before deployment. Six sequential checks follow the article’s argument: match the tool’s task to the deployment pathway; read the cleared indications and labeling, which define the breadth of the validated claim; demand external validation beyond the developing center; check that the validated device, operators, image quality, and population fit the intended setting; confirm institutional governance and monitoring (Figure 3); and disclose the AI’s contribution in the report and, where material, to the patient.
